# From sensor fusion to knowledge distillation in collaborative LIBS and hyperspectral imaging for mineral identification

**DOI:** 10.1038/s41598-024-59553-y

**Published:** 2024-04-20

**Authors:** Tomás Lopes, Diana Capela, Diana Guimarães, Miguel F. S. Ferreira, Pedro A. S. Jorge, Nuno A. Silva

**Affiliations:** 1 INESC TEC, Center for Applied Photonics, 4169-007 Porto, Portugal; 2 Departamento de Física, Faculdade de Ciências da Universidade do Porto, 4169-007 Porto, Portugal

**Keywords:** Optical spectroscopy, Scientific data

## Abstract

Multimodal spectral imaging offers a unique approach to the enhancement of the analytical capabilities of standalone spectroscopy techniques by combining information gathered from distinct sources. In this manuscript, we explore such opportunities by focusing on two well-known spectral imaging techniques, namely laser-induced breakdown spectroscopy, and hyperspectral imaging, and explore the opportunities of collaborative sensing for a case study involving mineral identification. In specific, the work builds upon two distinct approaches: a traditional sensor fusion, where we strive to increase the information gathered by including information from the two modalities; and a knowledge distillation approach, where the Laser Induced Breakdown spectroscopy is used as an autonomous supervisor for hyperspectral imaging. Our results show the potential of both approaches in enhancing the performance over a single modality sensing system, highlighting, in particular, the advantages of the knowledge distillation framework in maximizing the potential benefits of using multiple techniques to build more interpretable models and paving for industrial applications.

## Introduction

Spectral imaging is a research subject that uses spatially referenced spectral signatures to create informative visual maps of the surface of samples. The underlying motivation is that such spectral maps may contain valuable insight that largely expands the information acquired using traditional colored photographs, in specific leveraging on the detection capabilities of the spectroscopy technique utilized^1^. Indeed, a wide range of techniques have been investigated in the context of spectral imaging, including Laser-Induced Breakdown Spectroscopy (LIBS)^2^, Raman spectroscopy^3^, Energy Dispersive X-Ray Spectroscopy^4^, and hyperspectral reflectance imaging (HSI)^5^, which are now being actively incorporated in laboratories and industries for applications ranging from quality control to remote sensing^6,7^. In a distinct yet parallel direction, this strong market pull is now also fostering the opportunity to develop multimodal imaging solutions, where information from distinct sources is combined to enhance the capabilities of the individual system. Within this context, the development of algorithms and solutions that can capitalize on multimodality is a subject of paramount interest for science and technology^8–16^.

Focusing on the subject of mineral identification, LIBS and HSI are two of the techniques that have been extensively used in recent years. On one hand, LIBS is a spectroscopic technique that uses a focused, high-intensity laser beam to ablate the sample surface. Subsequently, the atomic species that constitute the sample may dissociate, excite and/or ionize, producing a plasma that starts to expand and decay, emitting radiation in the process that ranges from infrared to X-rays. In particular, the discrete lines of this spectral signature may be associated with specific transitions of atomic or ionic species, allowing us to obtain qualitative and quantitative information regarding the chemical composition of the sample. Expanding on this idea, LIBS imaging is made possible by sweeping the target surface in both transverse directions using a whisk broom technique, a concept that has shown gradually growing promise for both qualitative and quantitative examination of mineral samples^17–19^. Few and non-exhaustive examples include ore-grading^20,21^, mineral characterization^17–19,22^, and even historical studies using heritage-related samples^23^

HSI on the other hand, is a technique that gathers spectral data from the reflectance spectra of the target, typically from the visible to the short-wave infrared range (400nm to 3000 nm). In particular, the light radiation at specific wavelengths can be absorbed due to sub-molecular transitions, resulting in the bending and stretching of molecular bonds and leading to the appearance of dips in the reflectance spectrum that are called absorption bands. Being associated with specific molecular bonds, different minerals may reveal distinct spectral signatures, thus allowing to perform qualitative mineral identification and analysis. Compared with LIBS, one of the advantages of this technique is that it can explore a push broom scanning configuration, meaning that the map can be constructed by scanning the sample line by line, resulting in faster acquisition rates, a feature that is crucial for its applications in process control^24^ and aerial imaging^25,26^. Regarding mineral-related applications, HSI has found significant applications in mineral identification in particular using information in the NIR-SWIR imaging range^27,28^.

Still, while both LIBS and HSI are powerful tools individually, there are open challenges that still need to be overcome, including the reproducibility and inconsistency of results, instrumental drifts, and lack of interpretability^29^. Given these circumstances, approaches involving multimodal sensing are getting some traction within the scientific and engineering community, exploring the use of tandem solutions to workaround possible drawbacks of the individual techniques and increase the robustness and versatility of the systems^30^. Concerning multimodal sensing, the use of HSI in association with LIBS (LIBS-HSI) has been reported in the literature and typically explores the principle of data fusion. Depending on the approach, it can be called low-level fusion if entire datasets of different modalities are combined, mid-level fusion if only the extracted features are merged, and high-level fusion if it occurs at the decision level, i.e. utilizing the outputs of multiple models for each individual dataset and combining them to obtain a final classification. Some examples showcasing the capabilities of this approach include the classification of ginseng leaves according to plant species, geographical origin, and age using LIBS-HSI^31^, the classification and identification of rice geographical origins^32^, and the analysis of copper concentrates^33^. Overall, these results suggest that LIBS-HSI multimodal approaches show significant enhancements when compared to their standalone counterparts, exhibiting improved prediction capabilities along with better reproducibility, meaning that a multimodal approach could be of great relevance for multiple tasks such as mineral identification.

In this context, we intend to study the synergies of LIBS and HSI(and more specifically NIR-SWIR imaging), exploring two different strategies for collaborative sensing: (i) the more conventional mid-level sensor fusion approach, where we effectively extend the features available for identification by combining those extracted from the elemental (LIBS) and molecular (NIR-SWIR) composition; and (ii) a knowledge distillation framework, where we utilize an interpretable and unsupervised mineral identification methodology based on the LIBS modality^29^ to provide labels that are subsequently used to train the NIR-SWIR data, attempting to improve the classification using a supervised dimensionality reduction approach. Then, we describe

## Methodology

The major goal of this manuscript is to understand the capabilities of distinct sensing modalities and seek how to combine them in a multimodal spectral imaging solution that can increase performance over that of individual techniques. Besides, we also want to explore synergies that can leverage the individual advantages of the techniques and circumvent their drawbacks. Focusing on the specific context of LIBS-HSI multimodal spectral imaging solution for the purpose of this manuscript, we start this section by describing each technique and discussing its major advantages and drawbacks. We then advance to describe our approach to collaborative spectral imaging in two distinct directions. First, we focus on a more traditional sensor fusion approach, where we extract and combine features from the two techniques to train an unsupervised clustering algorithm for the identification of spatial regions of similar chemical content. Then, we describe a novel approach to collaborative sensing using a knowledge distillation framework, where the less-interpretable and noisier HSI modality is trained in a supervised manner using label predictions obtained from the LIBS technique.

**Figure 1:**
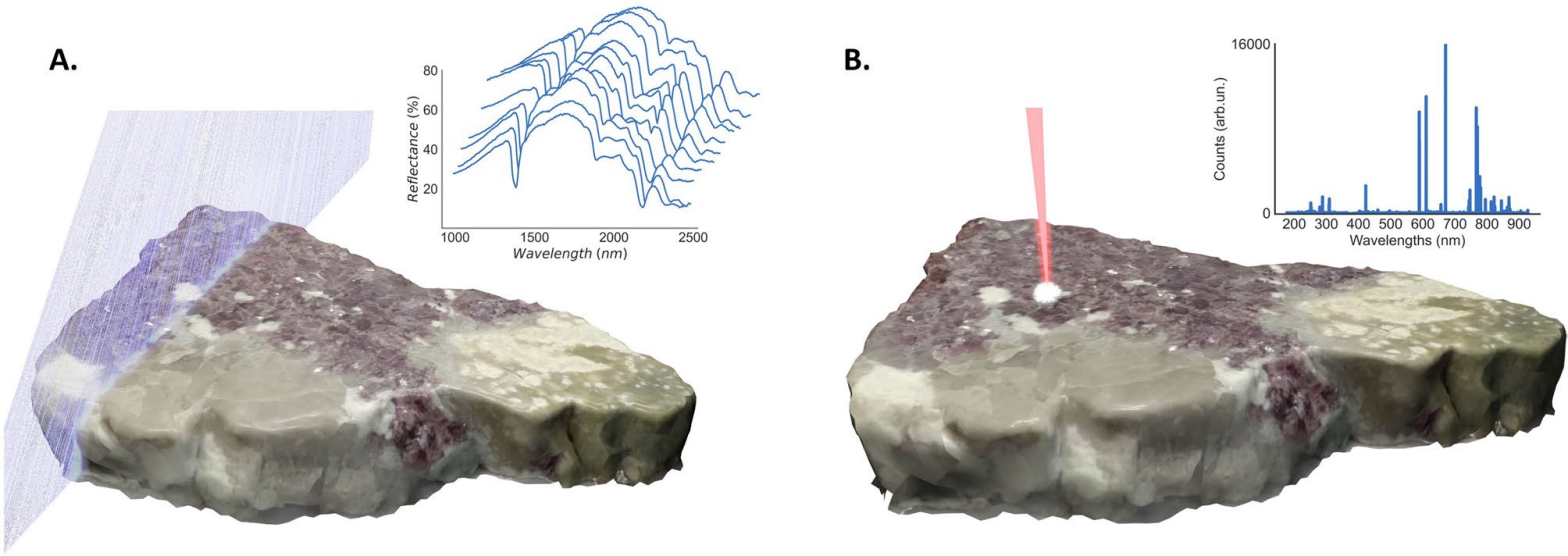
Illustration of the scanning strategy behind each technique and typical spectral information. With HSI (NIR-SWIR) (**A**), the dataset is constructed by scanning the sample using a line scan technique, while with LIBS (**B**) the data is generated by scanning the sample in a point-wise manner, leading to slower acquisition rates.

### Laser induced breakdown spectroscopy—LIBS

LIBS is a spectroscopy technique based on the analysis of multiple discrete emission lines obtained from a plasma decay. Compared with reference databases, the peaks obtained in the spectra and their intensity may be related to the presence and concentration of target chemical elements. For example, focusing on the geological samples, the differences in the characteristic spectral lines may assist in the identification of chemical elements at the sample surface, establishing a connection with the most probable mineral via its chemical composition. As this element analysis occurs at the focal spot, typically at the sub-millimeter scale, LIBS can be turned into a microscopic spectral imaging technique^34^ by scanning the sample surface in a point-wise manner (see Fig. 1) and using suitable numerical routines for signal processing and analysis^35,36^. Harnessing this power, a variety of tools have been developed and go as far as detecting minor compounds in complex rock samples^37^.

LIBS imaging features multiple advantages, from which we highlight its high dynamic range (most of the chemical elements present can be observed in a wide range of concentrations), high sensitivity (most of the time in the ppm range), information redundancy (multiple lines for each element), high spatial resolution (up to 10 $\upmu$m), and versatility (remote operation in harsh environments)^36^. For the drawbacks, we have the fact that even operating at 1KHz (typical systems work up to 100Hz), the whisk broom configuration translates itself into a rather slow technique, typically requiring hours to scan larger samples^38^. Furthermore, such high spatial resolution comes with a large amount of data generated, which also introduces challenges at the processing level, for which dimensionality reduction techniques such as PCA are often used to try to attenuate these problems^39,40^.

### Hyperspectral reflectance imaging—HSI

In turn, HSI is a spectroscopy technique based on the analysis of the surface reflectance from the visible to the infrared range. The goal is to observe a connection between the constituents of the sample and its optical properties, such as optical absorption, emission, reflection, and scattering. Relevant information may be contained in the location, the slope, the bending, and the depth of specific regions of the spectra, corresponding to some relevant molecules such as water and carbon trioxide^41^. Compared with LIBS, this technique allows for much greater speeds of acquisition when operating in the line-scan configuration (see Fig. 1), with a point acquisition rate that can reach 100 KHz, allowing for higher throughputs and making it suitable for industrial applications (see Table 1 for an overview of the parameters). Yet, in spite of this major advantage, HSI classification capabilities still underperform compared to LIBS due to its noisy (e.g. surface roughness) and convoluted information content, thus calling for novel approaches capable of enhancing this modality.

**Table 1:** Typical parameters for LIBS and HSI systems obtained and estimated from the literature and own systems.

Technique	Laser-induced breakdown spectroscopy imaging	Hyperspectral imaging
Scanning technique	Whisk broom	Push broom
Acquisition speed	1 ms per point	5 ms per line
Spectral resolution	0.1 nm	3–5 nm
Spatial resolution	100 $\upmu$m	400 pixels per line
Spectral range	200–900 nm	900–2500 nm (NIR-SWIR)

### The sensor fusion approach

Sensor fusion takes advantage of the fact that each technique may contain complementary information. Focusing on the mid-level sensor fusion approach, the aim is to combine them to effectively extend our feature space.

Our sensor fusion approach starts with the crucial step of aligning the spatial datasets for each modality. To achieve this, we select in each map a set of matching points^42^ before applying the Kabsch-Umeyama algorithm to find a suitable set of transformation parameters for the translation, rotation, and scaling of the datasets. This process, while simple to perform, requires immense precision in the matching point selection task to ensure proper alignment of the spatial features of each modality. Then, for this task, we looked for spectral maps for each technique that had resemblances between them, such as the sample outline and mineral transition regions to allow for a proper selection of a collection of matching points, so that the estimated transformation could get as close as possible to the ideal pixel-to-pixel match of the dataset and minimize the adverse effects of spectral image deformations of distinct modalities. Furthermore, given that a point-to-point match is necessary for fusion, we use the lowest resolution of LIBS imaging as the spatial mesh and associate each point with the closest point in NIR-SWIR imaging. Then, we proceed by treating each technique individually to extract the features at each point of the LIBS imaging spatial mesh.

**Figure 2:**
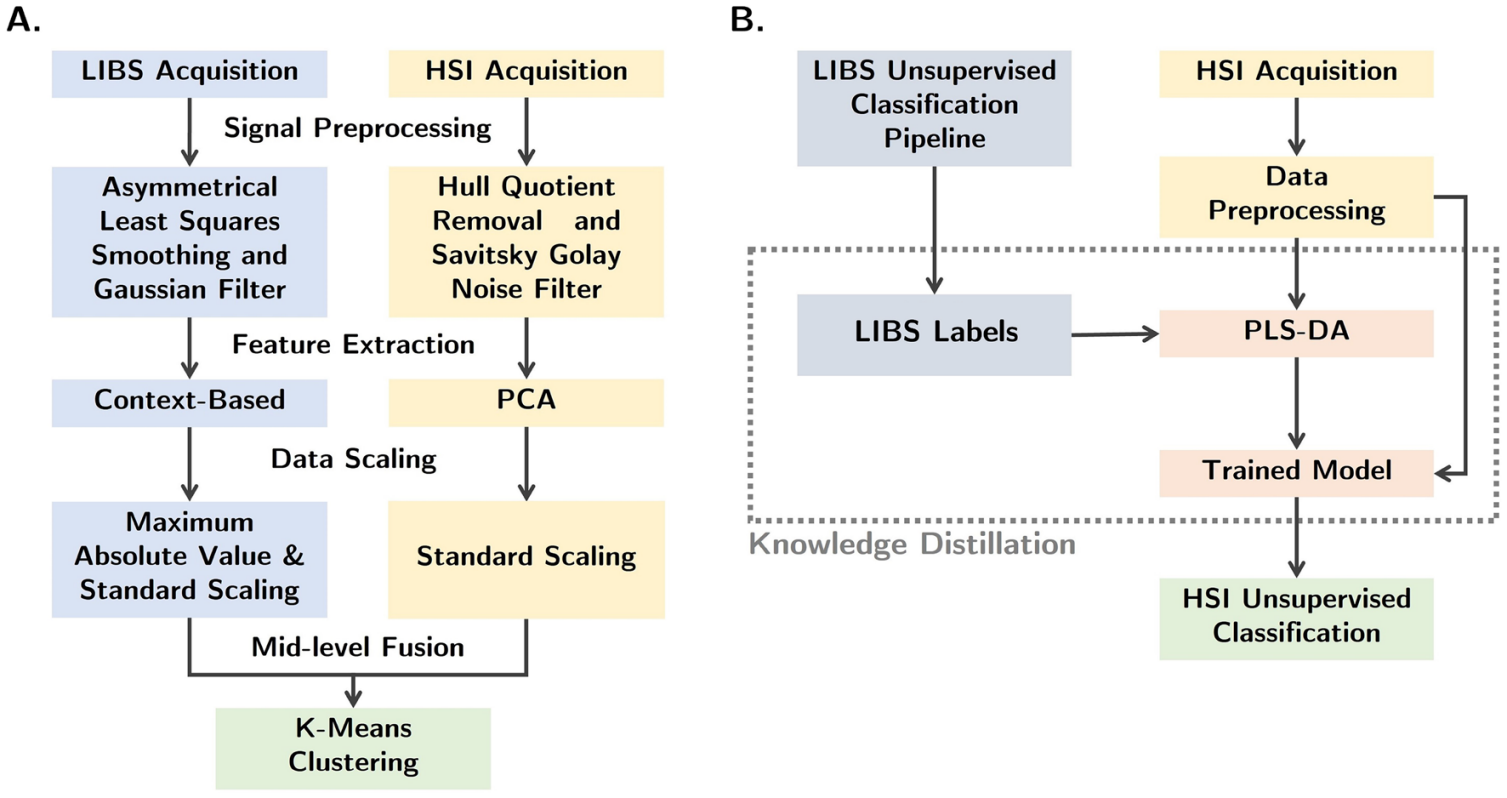
(**A**) Processing pipeline utilized for mineral identification using sensor fusion. (**B**) The proposed Multimodal Knowledge Distillation Pipeline utilized for mineral identification using LIBS soft labels to train a PLS-DA model taking NIR-SWIR data as its input.

In the second stage, each technique requires suitable pre-processing, followed by feature extraction and scaling before the mid-level fusion. For the case of LIBS imaging (see Fig. 2), the obtained signal contains not only the emission lines but also some background that results from Bremsstrahlung and recombination processes (continuous components). As this background has a non-constant spatial distribution that influences emission lines in a non-homogeneous way, its removal is a crucial step to achieve correct line intensities. This is achieved using a standard Asymmetrical Least Squares Smoothing algorithm^43^. Subsequently, a spatial Gaussian filter was also applied to decrease the influence of possible contaminations and minimize edge effects^29^. Following the preprocessing step, feature selection is performed using a context-based approach, that selects wavelengths of interest according to our prior knowledge of possible elements that constitute the sample (more details on the technique can be found in ref.^29^). Finally, each feature is scaled to its maximum absolute value, assuring in the process that we preserve the shape of the distribution. In particular, in direct comparison with a block-scaling methodology, we note that this allows us to preserve the importance of each element and emission line for the final classifier independent of their relative values. For fusion, additional scaling is applied in the form of standard scaling to prevent any kind of bias of distinct modalities.

For NIR-SWIR, the raw spectra obtained from the camera device are first normalized to a white reference to obtain reflectance spectra, revealing characteristic absorption features as well as characteristic background signatures. This background signature is, in part, a consequence of ferrous ions, water, and carbonate absorptions that are outside the spectrometer range^41^. While it can be argued that this so-called reflectance hull may provide additional spectral information, its curvature tends to distort the spectral absorption features, and as such, its removal is desirable. Therefore, we start our processing pipeline for NIR-SWIR data by applying a Savitsky-Golay filter to remove noise, followed by hull quotient correction to remove the reflectance hull (see Fig. 2). Contrary to LIBS, context-based extraction is harder in NIR-SWIR^41^. Taking this into consideration, we used a conventional PCA analysis for dimensionality reduction, selecting the first four principal components that account for an explained variance ratio of 98%. Taking the scores as the extracted features, a standard scaling is then applied for the same reasons discussed previously.

Having the features extracted for each model, we concatenate them in a mid-level fusion stage (feature-level fusion) into a single dataset that can then be used to train an unsupervised classification algorithm. For this case, and inspired by previous results for LIBS^29^, we have chosen to utilize conventional K-means clustering. The algorithm is first trained for a reference sample that we use to interpret the results and label the clusters (i.e., assign to mineral type) before generalizing it to unseen samples as a Rocchio classification. Finally, we compare the results obtained using collaborative sensing with those for the standalone techniques, i.e., trained only with the extracted features for each technique.

The described computing workflow was implemented using Python routines together with the libraries *numpy*, for array manipulation, *scikit-learn* for machine learning and *spectral* for the NIR-SWIR data preprocessing.

### A multimodal knowledge distillation approach

In the context of machine learning, knowledge distillation is, in general terms, a process of condensing and transferring knowledge from a complex model to a simpler one. Inspired by this concept, a Cross-Modal knowledge distillation (CMKD) was recently proposed in the literature^44^ in particular for RGB images. In short, using a *Teacher–Student* scheme, the CMKD takes advantage of excellent performances, provided by a modality with superior knowledge (the Teacher), and transfers it to a weaker modality that, on its own, provides lower performances (the Student). Yet, the concept of CMKD is usually applied to neural networks and often relies on the supervised training of the teacher modality.

For the context of this work, we suggest a novel approach to this concept in the form of *Multimodal Spectral Knowledge Distillation* (MSKD). In MSKD, the workflow(see Fig. 2) starts with the training of a classifier using a single spectroscopy modality in an unsupervised manner, subsequently using the output of this classifier as a label to train the weaker spectroscopy modality with a suitable supervised learning algorithm. Put in this way, we believe that MSKD may offer non-trivial advantages for multimodal spectral imaging, in particular, because it exploits the superior performance of supervised learning^45^ while bypassing the laborious (and often imprecise) work of hand labeling correct pixel regions. Indeed, this work can be substituted with a simpler cluster label assignment task which can leverage the interpretability of the first technique, making this design particularly promising for intelligent online industrial applications.

To get into further details, we can consider our case study on mineral identification using LIBS and NIR-SWIR imaging. In principle, applying supervised learning to NIR-SWIR imaging will allow an increase in the performance over PCA-based unsupervised clustering while mitigating the effects of noise and circumventing the lack of interpretability of NIR-SWIR data. Our goal is to make use of the superior interpretability and performance of LIBS alone (identification of elements allowing for robust and interpretable results from unsupervised clustering techniques^29^) as the teacher to train a NIR-SWIR pipeline, the student. Having the soft labels, we chose a supervised learning algorithm suitable for this task. Our choice was to explore Partial Least Square Discriminant Analysis (PLS-DA) due to its proven effectiveness in working with hyperspectral data^46^, correctly dealing with multi-collinearity in dimensionality reduction tasks related with the task in hand.

## Results and discussion

**Figure 3:**
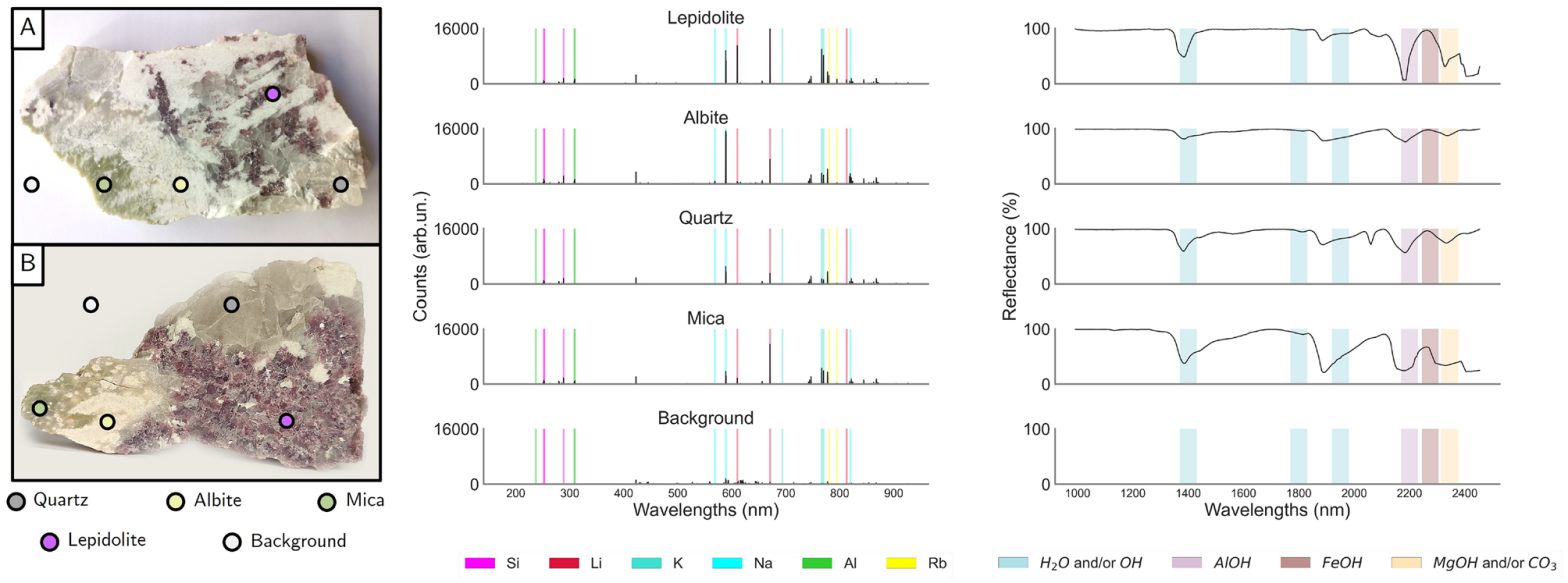
(**A**) Rock samples and indicative mineral zones. Spectral signature for (**B**) LIBS and (**C**) NIR-SWIR, showcasing the typical signature of distinct mineral types.

To test our collaborative sensing strategies and mineral identification pipelines, two rock samples obtained from the same mining site were selected, as seen in Fig. 3A, both exhibiting a similar mineralogical composition. The samples are fragments of a Li-rich pegmatite vein with economic potential for mining exploration, located in the Central Iberian Zone of the Iberian Massif. From previous studies^47^, it is known that these are mostly composed of 4 mineral types, namely *Lepidolite*, *Quartz*, *Albite*, and *Mica*. Using reference chemical formulas (see Table 2, including Rubidium (Rb) as a proxy indicator)^48^, we can select the elements of interest (EoI) to be used for feature extraction in LIBS. The relevance of this case study is two-fold. On one hand, the samples belong to a well-studied location with an economic interest in the context of lithium mining, which not only eases the process of analysis but also demonstrates the potential of the approach for a real-world technological application, allowing to test generalization capabilities. On the other hand, the minerals can be identified by their distinctive color(in most zones of the samples), allowing to compare with the expected prediction: Lepidolite (purple), albite (white), quartz (grey), and Li-mica (light green–grey).

For the purpose of this work, we have used a prototype LIBS system consisting of a Nd:YAG laser, operating at a repetition rate of 1Hz, with plasma emission being captured by eight spectrometers operating in the range of 200–900 nm. The laser pulse energy was set to 47.5 mJ, with the spectrometers operating with a gate delay and integration time of 1.3 $\upmu$s and 1.05 ms, respectively. The NIR-SWIR data was collected using a Specim SWIR hyperspectral camera covering a range that spans from 1000–2500 nm, with a resolution of 384 pixels per line. The camera acquired the data in a horizontal acquisition, and the scan was performed using an additional conveyor belt of 40 cm of width, and velocity close to 10 cm/s.

### Sensor fusion

To evaluate the results obtained in sensor fusion, we start by analyzing the spectral signature of each mineral for both LIBS and NIR-SWIR as seen in Fig. 3B (refer to the included supplemental material for a more comprehensive analysis of the extracted features). As expected, the LIBS spectrum features variations in spectral line intensities corresponding to elements present in each mineral. These correspond to Lithium (Li), Silicon (Si), Potassium (K), Sodium (Na), Aluminium (Al), and Rubidium (Rb). As such, these were the lines used for our context-based feature extraction step (see Table 3 for the complete list of lines).

Turning our attention to NIR-SWIR imaging, each mineral is no longer associated with specific lines, but rather with distinct bends and dips of the reflectance curves, with the main sources of variability now originating from different slopes and depths of bands in the spectra. In this context, using a conventional PCA for dimensionality reduction is a common approach for feature extraction^49^. In this methodology, the set of component scores becomes our features, and we can rely on the loadings to provide us with some degree of interpretability, and understanding how the components are related to the original feature space. The number of components to be used in the PCA method was obtained by analyzing the scree-plot of the explained variance, setting a threshold of 98%, which is achieved using four components.

**Figure 4:**
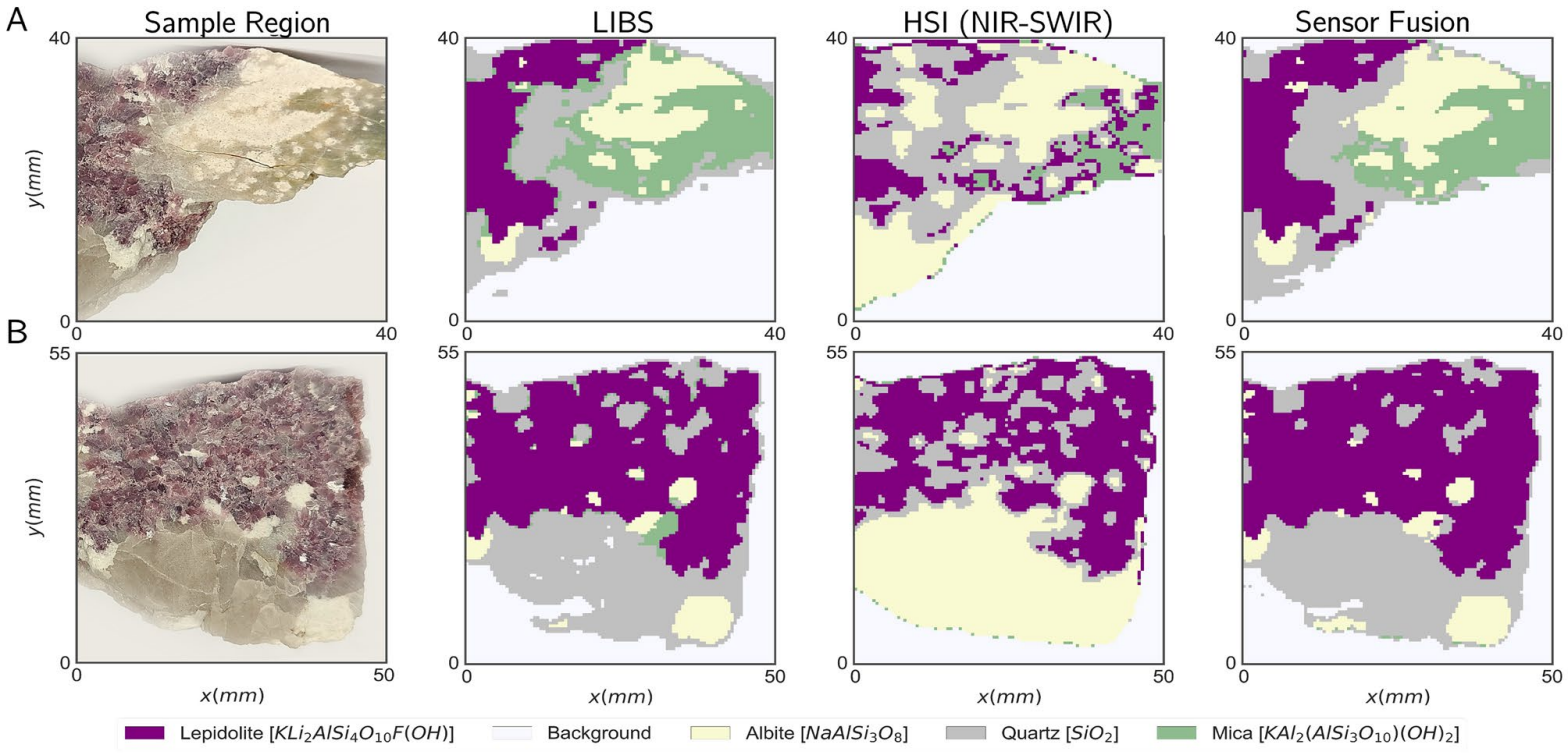
(**A**) Classification results were obtained by applying our training pipelines to a training region. (**B**) Classification results obtained by applying our trained models to a test sample region.

Having a brief overview of how we expect each technique to provide differentiation of mineral regions, we can advance to deploy the actual mineral classification algorithm. For this, and for simplicity of operation, we opt to train a K-means unsupervised clustering algorithm, comparing the results obtained for each standalone method to the sensor fusion approach seen in Fig. 4.

For the training stage, we started by choosing a suitable training set, opting for the region seen in Fig. 4A. In this region, the minerals appear to be better defined, which will be instrumental to better interpret the results qualitatively. Furthermore, the dataset seems well-balanced in terms of mineral area, which prevents the appearance of unwanted bias. The next task of the training stage is to choose the number of clusters to be used during training. For the purpose of this work, we relied on prior expert analysis of the sample, e.g. confirming the presence of four major mineral regions plus the background, thus totaling five clusters. Alternatively, in the absence of this expert analysis, one can still analyze how the total cluster inertia varies with the number of clusters, estimating the ideal cluster number using an empirical *elbow method*^29,50^.

**Figure 5:**
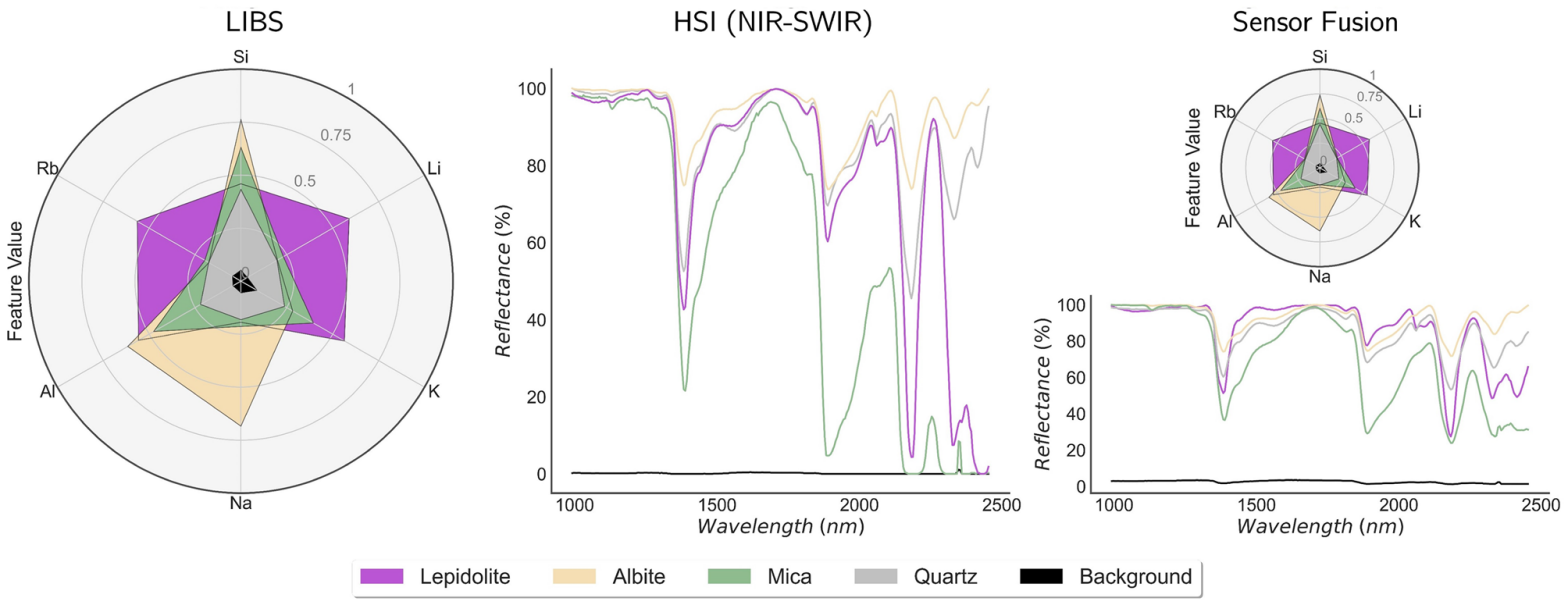
Perceptual map exhibiting the average LIBS feature value and average NIR-SWIR spectrum for each of the identified clusters. In each case, various degrees of interpretability can be achieved, with LIBS providing a higher degree of interpretable information.

We shall note that having the clusters identified after the training stage only groups surface zones in mineral types but does not provide a prediction of which mineral it corresponds to. Indeed, we need an additional label assignment stage^29^, in which we assign each cluster to the corresponding mineral type. To achieve that we can proceed with a user interpretation of the results at the end of the training stage. For this, we calculate the centroids in the feature space, and present the results in an informative graphical format, as in Fig. 5. Now, using the radar chart for the LIBS feature space, it is easy to associate the cluster with the centroid with non-zero features in the elements of interest, according to Table 2. For example, the cluster with Li and Rb features shall be clearly associated with the Lepidolite mineral in this context. For the NIR-SWIR only, one can still obtain the centroid and recover it onto the original feature space, obtaining a spectrum that can be compared against a reference one, in particular comparing to those in Fig. 3. While this qualitative step can be trickier to perform than its LIBS counterpart, we expect it to be sufficient for a correct interpretation of some mineral types^41^.

Another way to approach the label assignment task is to provide our own cluster center initializations. To achieve that, we can start with an estimate of the feature values for the centroids of each mineral cluster. For the LIBS case, the context-based feature extraction turns this into a trivial task: we can initialize the centroid values with 1 for lines we expect to be present, and 0 for those we are not, according to Table 3 (e.g. for Quartz we initialize the centroid with 1 for Si lines, and 0 for all the others). However, this process turns out to be exclusive to the LIBS feature space as the PCA components for NIR-SWIR do not allow the same degree of interpretability. Still, we can apply this methodology to the sensor fusion approach, initializing the LIBS features and providing random initialization to the NIR-SWIR features, which converges to the expected results for the present case study.

**Table 2:** Chemical formula of the four most relevant minerals observed in the samples.

Mineral	Chemical composition
Lepidolite	${\rm KLi}_2{\rm AlSi}_4{\rm O}_{10}{\rm F(OH)}$
Albite	${\rm NaAlSi}_3{\rm O}_8$
Quartz	${\rm SiO}_2$
Mica	${\rm KAl}_2({\rm AlSi}_3{\rm O}_{10})({\rm OH})_2$

**Table 3:** Elemental lines selected for the feature extraction procedure applied to the LIBS dataset.

Element	Emission Lines		
Al	237.23	308.17	309.25
K	693.80	766.40	769.81
Li	610.22	670.76	812.56
Na	568.77	588.95	819.40
Si	288.16	251.59	252.82
Rb	779.94	794.58	–

Analyzing the results obtained for the training dataset (Fig. 4A), it is straightforward to conclude that LIBS and NIR-SWIR imaging provide very distinct results. Starting with LIBS, we can see that the results are in good accordance with the expected from the sample figures, noting some incorrect classification of Quartz and Mica adjacent regions in sample B, along with some mineral transition zones. These edge effects have been reported in literature^29^ and come to be expected for LIBS-based imaging since, depending on the crater size (spatial resolution), the mineral boundaries often contain information regarding more than one mineral, introducing some ambiguity in the model. Regarding NIR-SWIR, it is clear that the PCA-based method has a poor performance, having very little agreement with the mineral regions in the training and test samples. Finally, our sensor fusion approach seems to provide a good agreement with the sample figures, possibly overperforming LIBS-only classification as it seems to eliminate edge effects. Besides, Mica and Quartz transitions also present signs of improvements, with Quartz regions previously unidentified in LIBS being now present (see Fig. 4A).

Having a trained model we can go a step further and use it to automatically identify minerals in distinct samples of the same mineralogical composition as is the case of the test region seen in Fig. 4B. The results obtained further demonstrate that for both LIBS and sensor fusion, there is a strong correlation between the expected mineral distribution and the results of the clustering algorithm, with sensor fusion again reducing edge effects and even enhancing the distinction of Albite and Mica regions. Hyperspectral imaging shows again that it underperforms when compared with the other approaches, as it is only able to find the outline of the entire sample and correctly identify some Lepidolite regions.

Finally, in addition to the obtained results, one shall also mention that sensor fusion may provide not only better performance but also a higher degree of interpretability. Indeed, by averaging both the NIR-SWIR spectra and LIBS emission lines at the cluster regions, yielding the centroid composition (see Fig. 5), we obtain both an average composition in terms of elements present using the LIBS radar chart and average absorption spectra for each mineral type using NIR-SWIR modality. In principle, with some expert knowledge, this may ease the cluster labeling stage and increase its interpretability, providing a higher degree of information that can be cross-checked.

### Multimodal spectral knowledge distillation

From the analysis of the results in the previous section, it becomes clear that NIR-SWIR data with the classical PCA-based methodology underperforms in the task of mineral identification. However, from the fact that distinct minerals contain distinct reflectance signatures as seen in Fig. 3, one can argue that this is not caused by poor information content but rather related with the intermediate steps and feature extraction methodology. Indeed, with PCA dimensionality reduction, one looks to preserve most of the variance present in the original dataset (say around 90 to 95%), disregarding low-variance components. While usually the latter contain noise-related information, it may also happen that significant information is enclosed in smaller variations and thus neglected and lost during the dimensionality reduction process. Thus a supervised dimensionality reduction methodology would be far more suitable for avoiding such troubles.

**Figure 6:**
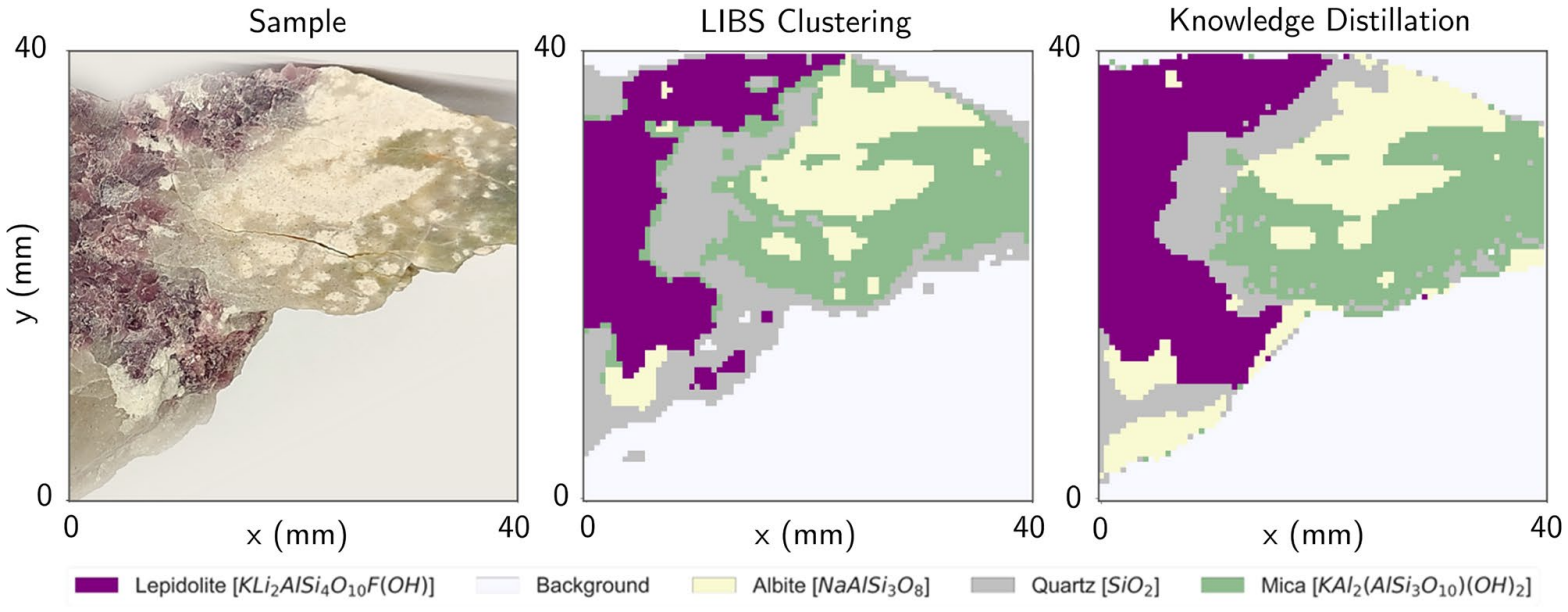
Unsupervised classification results of standalone LIBS, and resulting classification provided by the knowledge distillation pipeline that takes the LIBS classification as labels.

**Figure 7:**
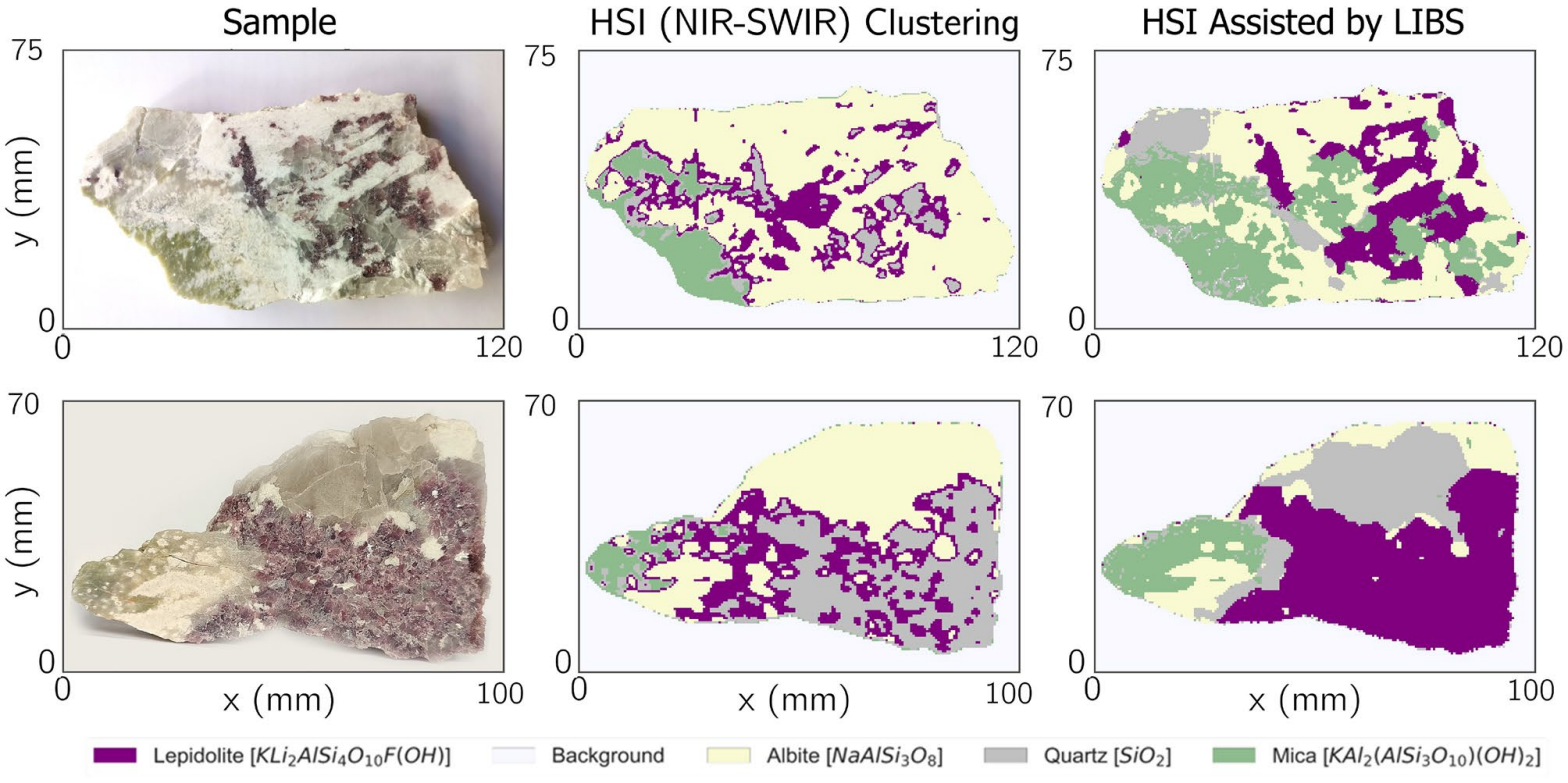
Comparison of the classification results between standalone NIR-SWIR imaging and the knowledge distillation pipeline on test samples.

This context sets a particularly interesting scenario to use the multimodal spectral knowledge distillation pipeline we propose in this manuscript, using a superior knowledge technique - LIBS - to train the NIR-SWIR imaging technique. Using the same training set as in the previous section, we utilize the previously trained K-means method in the LIBS imaging dataset only to generate labels for the train dataset. We then proceed with the knowledge distillation step, using a PLS-DA for the supervised learning algorithm on the NIR-SWIR data only. To determine the optimal number of components for the PLS-DA the predictive error analysis on the train sample was calculated using the LIBS labels as the ground truth, being careful to prevent model overfitting. Taking these criteria into consideration, we have selected 17 components for this case study.

The results obtained are depicted in Fig. 6. First, taking the LIBS labels as the ground truth, it is straightforward to conclude that MSKD significantly enhances the performance over an unsupervised NIR-SWIR classification presented in Fig. 4B. Besides, the NIR-SWIR-only classifications with MSKD are now also on par with those obtained with LIBS.

More interestingly, one can now utilize the trained model and generalize to unseen samples as presented in Fig. 7. Comparing with empirical knowledge about the samples (color) and further expert validation, the results enclosed show that although some cluttered regions in sample A and borders in sample B do present some erratic classification, in particular of Quartz regions, the overall performance has significantly improved when compared to the standard PCA and K-means unsupervised pipeline. This suggests that the unsupervised LIBS knowledge was able to train a model in the NIR-SWIR domain, enhancing its capabilities by acting as an autonomous supervisor. Furthermore, we highlight that these results are achieved in the NIR-SWIR imaging dataset only, meaning that no further information from LIBS is required, which presents a major advantage over typical sensor fusion approaches, allowing them to achieve similar performances at higher throughputs.

Edge effects of LIBS may hinder the resulting NIR-SWIR model from the knowledge distillation procedure as the success of the strategy is intrinsically tied to the correct classification of the teacher technique. Yet, contrary to what happens in typical knowledge distillation frameworks, this is not directly connected with a strong limitation to the overall performance. Indeed, as the information source is not the same, and the number of free parameters in our model is lower than the size of the training dataset thus preventing overfitting, the NIR-SWIR model may still correctly learn the distinctive features to classify the samples that in a configuration of higher resolution, may allow to solve edge effects. This interesting take is one of the opportunities that spectral knowledge distillation offers and its impact makes it an interesting challenge for future research on the topic with carefully designed experimental procedures for that specific purpose.

## Concluding remarks

In this manuscript, we explored analytical strategies in the context of multimodal spectral imaging, aiming to efficiently exploit the synergy of two spectroscopy techniques. More specifically, the work focused on two distinct approaches, featuring distinct characteristics and advantages. On one hand, we explored a traditional sensor fusion approach, combining data of both sensing modalities at the feature level and deploying an unsupervised classifier by performing clustering in this augmented feature space. On the other hand, we proposed an innovative knowledge distillation approach, that leverages the accuracy and robustness of a sensing modality—the *teacher*—to generate labels for a training dataset, subsequently feeding the supervised training procedure using the dataset of the second spectral imaging modality—the *student*. For the purpose of the work, we introduced a case study with LIBS and HSI (NIR-SWIR) imaging to perform a mineral identification task.

The results obtained demonstrate that when considering the standalone approaches, LIBS has a clear advantage in classification capabilities, with NIR-SWIR imaging struggling to correctly identify the target minerals. Combining the data from both sensing modalities using a mid-level fusion architecture did improve slightly the performance when compared to LIBS, in particular mitigating boundary artifacts and possibly improving interpretability by direct analysis of the cluster centroid. The results suggest that such a sensor fusion approach may be an interesting approach for applications where higher degrees of accuracy are necessary or when a technique, e.g. LIBS, partly struggles due to the complexity of the matrix.

Advancing to the multimodal spectral knowledge distillation strategy, we were able to utilize this novel approach to successfully train a model that takes only NIR-SWIR data as input using the LIBS technique as a supervisor during training. Although distinct, this comes with significant advantages. On one hand, it allowed us to obtain significantly higher classification accuracies of the hyperspectral technique when compared to its standalone counterpart, setting the opportunity to use a spectral imaging technique as an autonomous supervisor for the other. On the other hand, we must also emphasize that such an approach allows us to effectively capitalize multimodality, exploiting the benefits of single-modality systems to deploy a solution of higher performance. For the example discussed, leveraging on the NIR-SWIR imaging versatility and higher throughput compared to LIBS, the final solution is able to preserve the higher performance of LIBS at the resolution and operation speed of the HSI solutions.

Overall, the findings enclosed open new perspectives for the subject of spectral imaging both at the academic and technological levels. In particular, future research directions can take advantage of the knowledge distillation pipeline to deploy efficient industrial applications, where high throughput and robustness are desirable. Besides, taking into consideration that multimodality is only required at the training stage, also holds the potential to reduce the running cost of such systems, requiring only a single spectral imaging modality during the operation. Finally, although the focus of the manuscript was on the mineral identification procedure, the findings enclosed can be straightforwardly generalized for other research fields and classification tasks in the vast subject of spectral imaging (Supplementary Information).

### Supplementary Information

Supplementary Information.
