# Supplementary material for: From sensor fusion to knowledge distillation in collaborative LIBS and hyperspectral imaging for mineral identification
Source: Sci Rep. 2024 Apr 20;14:9123. doi: 10.1038/s41598-024-59553-y (PMC11032373; doi:10.1038/s41598-024-59553-y)
Supplement: Supplementary file 1 — Supplementary Information. [file 41598_2024_59553_MOESM1_ESM.pdf]

Supplementary Material: From sensor fusion to knowledge distillation in collaborative LIBS and Hyperspectral imaging for mineral identification

The supplemental material included in this document further supports the results provided in the original manuscript.

1. MAPS OF LIBS FEATURES

Maps for the utilized LIBS features are presented in S1 and S2. It becomes noticeable that no element has a corresponding exclusive map, i.e., no region of the sample is present in a single map, meaning that an immediate qualitative distinction among all the mineral regions is not possible. That is expected since in LIBS analysis we are looking at the presence of specific elements and many mineral in nature share elements in their composition. A particular example is silicon, which is present among nearly all mineral types.

Yet, it also becomes apparent that clustering of the mineral regions using these features is possible by looking at specific combinations of these element maps, which is precisely what k-means clustering is looking for, and is one of the reasons why it performs so well.

2. MAPS OF NIR-SWIR FEATURES

Turning our attention to the reflectance NIR-SWIR maps (see Figures S3 and S4), we notice again that there are no exclusive maps. Contrary to LIBS, these are not related to elements in the sample but to absorption bands that appear caused by molecules present in the sample, and when one looks into some of the most common ones, those being H_20, $AlOH$, $FeOH$, $MgOH$, and CO_3, there are not any clear distinctions between them. Then, it becomes clear that perhaps a simple feature selection of features procedure for these techniques might not be as suitable as for the LIBS case, and, as such, other feature selection methodologies need to be applied, as was the case of our PCA dimensionality reduction procedure. However, as seen from the results in the manuscript, that was still not enough to allow this technique to achieve an accurate classification performance

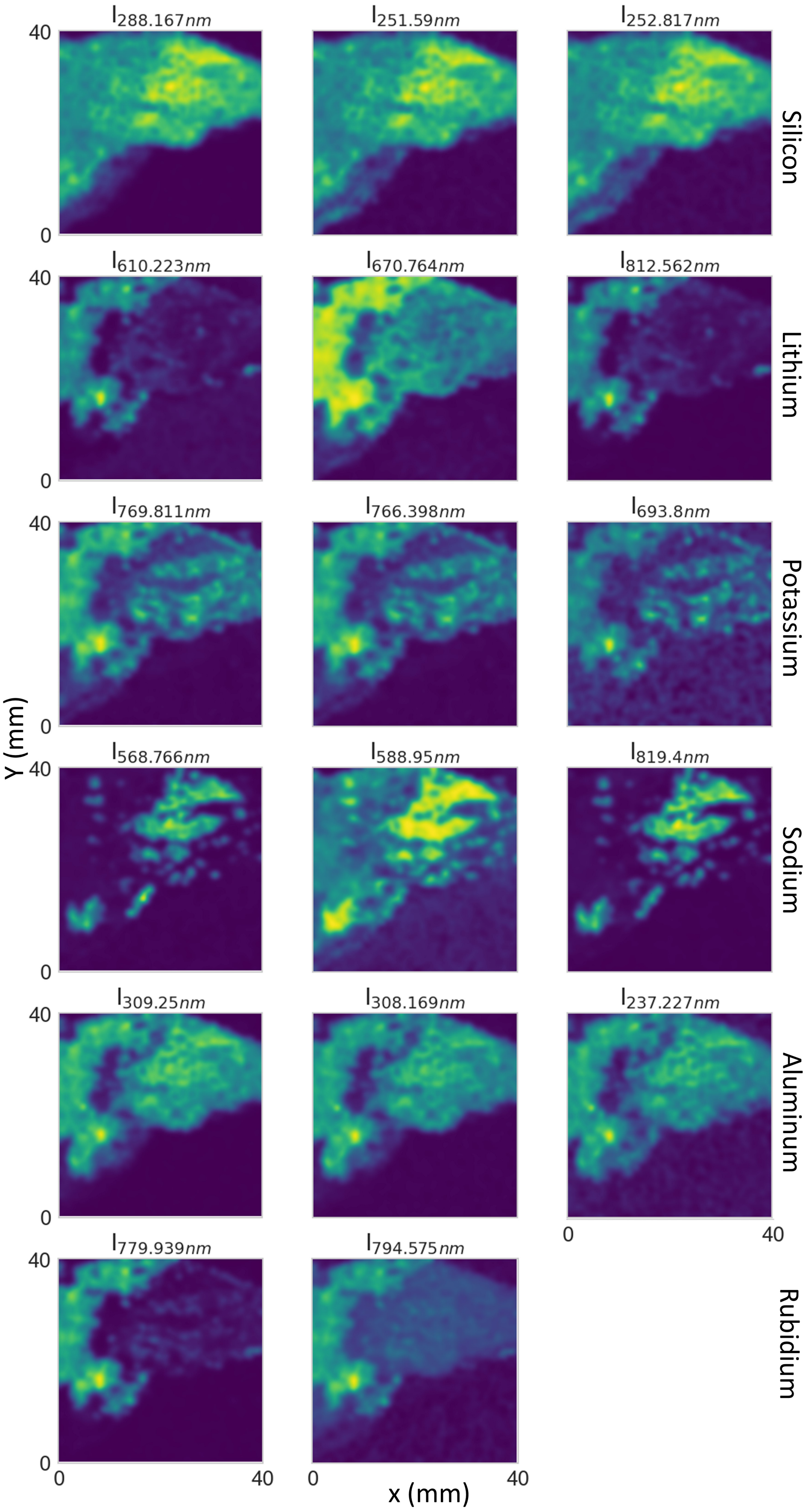

Fig. S1. LIBS maps of the train dataset for the emission lines selected in the feature extraction procedure for the various elements that constitute the sample at study

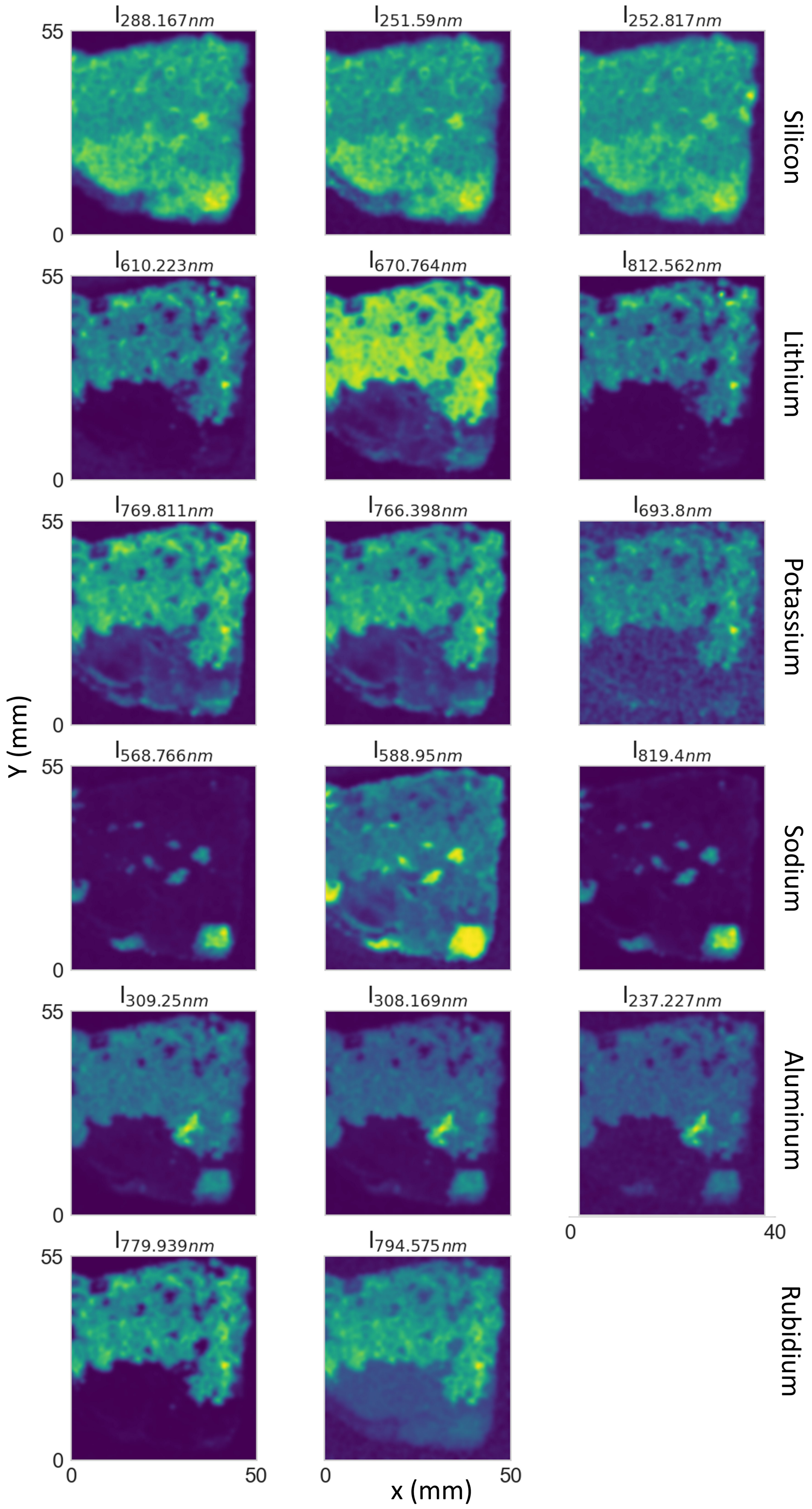

Fig. S2. LIBS maps of the test dataset for the emission lines selected in the feature extraction procedure for the various elements that constitute the sample at study

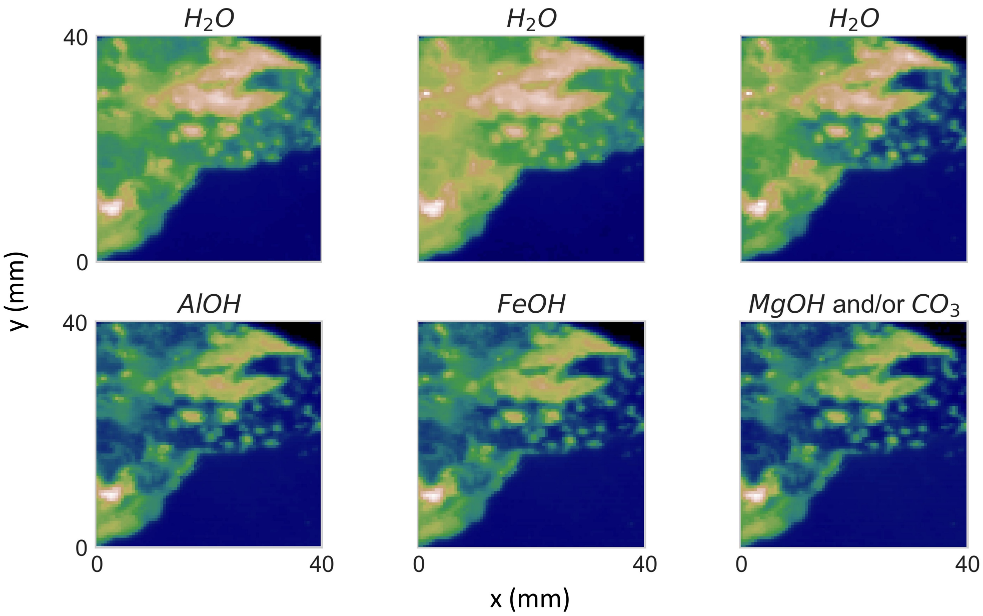

Fig. S3. NIR-SWIR maps of some of the most common bands seen in the mineralogical sample for the train dataset

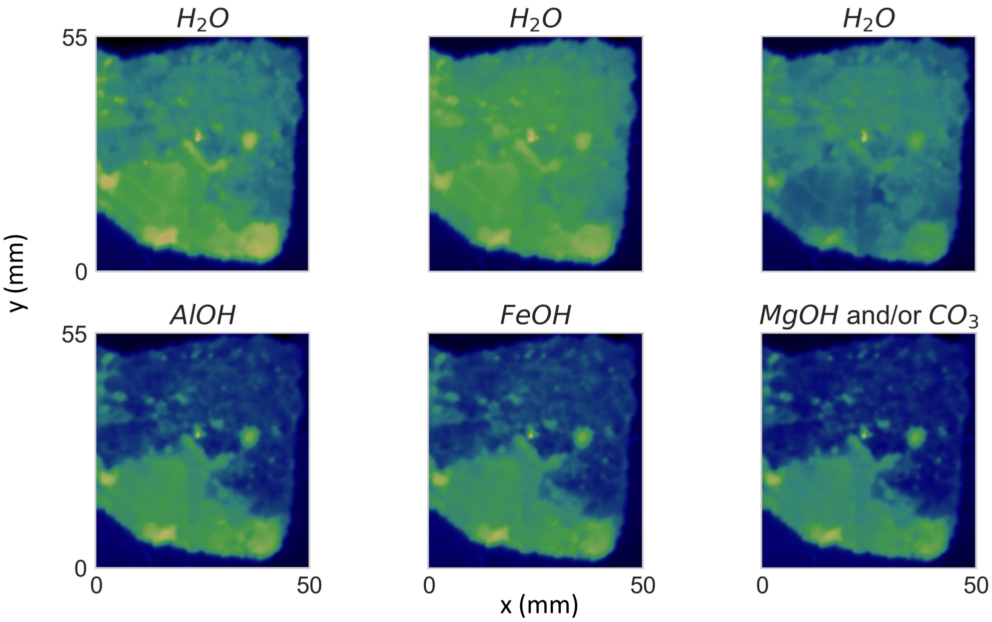

Fig. S4. NIR-SWIR maps of some of the most common bands seen in the mineralogical sample for the test dataset
